# Draft Genome Sequence of Enterobacter hormaechei subsp. *steigerwaltii* Strain BEI01

**DOI:** 10.1128/MRA.00406-21

**Published:** 2021-07-15

**Authors:** Soon Keong Wee, Sharon Cui Mun Chan, Xue Li Guan, Eric Peng Huat Yap

**Affiliations:** a Lee Kong Chian School of Medicine, Nanyang Technological University, Singapore, Singapore; Loyola University Chicago

## Abstract

Here, we report the genome sequence of Enterobacter hormaechei subsp. *steigerwaltii* strain BEI01, originally deposited as a member of the Enterobacter cloacae complex. The genome is 4,900,246 bp in size with a GC content of 55.44%; it contains multidrug antimicrobial resistance genes and several metal resistance gene operons.

## ANNOUNCEMENT

The Enterobacter cloacae complex (ECC) comprises six different species, namely, E. cloacae, E. asburiae, E. kobei, E. hormaechei, E. ludwigii, and E. nimipressuralis, that can cause a range of nosocomial infections (1). As part of the *Enterobacteriaceae* family, it is also capable of acquiring antimicrobial resistance (AMR), leading to the emergence of multidrug-resistant isolates. Species identification of these opportunistic pathogens allows better molecular epidemiology study of its transmission and AMR. Here, we report the draft genome sequence of Enterobacter hormaechei subsp. *steigerwaltii* strain BEI01, which was revealed to be a multidrug-resistant strain with remarkable antimicrobial and metal resistance genes.

Enterobacter cloacae complex strain BEI01 was obtained through BEI Resources, catalog number NR-50391. The isolate was grown overnight in lysogeny broth (LB) at 37°C with aeration. Genomic DNA extracted using a QIAamp DNA minikit (Qiagen, Germany) was sequenced using the Nextera XT DNA library preparation kit (Illumina, USA). A total of 1,594,314 sequencing reads from an Illumina MiSeq run using 300-bp paired-end sequencing were obtained for *de novo* assembly using Unicycler (Galaxy 0.4.8.0) (2). Annotation of the 69 contigs (coverage depth, 57-fold; *N*_50_, 349,141 bp) was performed using the NCBI Prokaryotic Genome Annotation Pipeline 5.1 (3). Species identification was confirmed using the Type (Strain) Genome Server (4) and average nucleotide identity (ANI) calculator (5). Multilocus sequencing typing (MLST) using the Enterobacter cloacae typing scheme was conducted on PubMLST (6). Antimicrobial and metal resistance genes were predicted from protein sequences using NCBI AMRFinderPlus 3.9.8. (7). Default parameters were used for all software tools.

Enterobacter hormaechei subsp. *steigerwaltii* BEI01 has a genome size of 4,900,246 bp with a GC content of 55.44%. The draft assembly contains 4,600 coding DNA sequence (CDS) genes, 73 pseudogenes, 79 tRNAs, 5 rRNAs, and 5 noncoding RNAs (ncRNAs). Four intact prophage regions (69.8 kb, 57.8 kb, 44.4 kb, and 43.6 kb), with three questionable and two incomplete prophage regions, were predicted using PHASTER (8). No CRISPR arrays were detected using CRISPRCasFinder (9). The isolate belongs to sequence type 93 (ST93), a global clone of carbapenemase-producing Enterobacter species (10). Although the bacterium was originally deposited as a member of the Enterobacter cloacae complex, digital DNA-DNA hybridization (dDDH) revealed that the genome belonged to the species Enterobacter hormaechei subsp. *steigerwaltii* with a 91.2% dDDH_4_ (95% confidence interval, 89.0 to 92.9%) match (dDDH_4_ is the dDDH value using distance formula *d*_4_). An ANI value of 98.99% was calculated using *E. hormaechei* subsp. *steigerwaltii* strain DSM 16691 (GenBank accession number CP017179.1) as the reference genome. *In silico* detection of plasmids using PlasmidFinder 2.0 identified two contigs harboring the IncN and IncR plasmid types (11).

Enterobacter hormaechei subsp. *steigerwaltii* BEI01 is a multidrug-resistant isolate containing 10 antimicrobial resistance genes (ARG) against 6 classes of antimicrobials, as well as 20 metal resistance genes (MRG) against 3 different metals (Table 1). The presence of *bla*_KPC-2_ highlights the significance of this isolate as part of the ST93 clonal group that is known to carry carbapenemase-producing genes. MRG against arsenic, mercury, and silver were observed to be located in close proximity to operons in their respective contigs. This suggests the need for further investigation of MRG and their potential role in allowing bacteria to persist in the hospital or the natural environment.

**TABLE 1:** List of antimicrobial resistance genes and metal resistance genes present in Enterobacter hormaechei subsp. *steigerwaltii* BEI01

Class	Gene(s)
Antimicrobial resistance genes	
Aminoglycoside	*aadA1*, *aac(6′)-Ib′*
Beta-lactam	*bla*_ACT-17_, *bla*_KPC-2_, *bla*_OXA-9_, *bla*_TEM_
Fosfomycin	*fosA*
Phenicol	*catA*
Quinolone	*oqxB*
Trimethoprim	*drfA14*
Metal resistance genes	
Arsenic	*arsC*, *arsB*, *arsA*, *arsD*, *arsR*
Mercury	*merE*, *merD*, *merA*, *merC*, *merP*, *merT*, *merR*
Silver	*silE*, *silS*, *silR*, *silC*, *silF*, *silB*, *silA*, *silP*
Others	
Acid resistance	*asr*
Efflux pump	*fieF*
Siderophore	*iroN*

### Data availability.

This whole-genome shotgun project has been deposited at DDBJ/ENA/GenBank under the accession number JAFRUD000000000. The version described in this paper is JAFRUD010000000. The isolate has been deposited under BioSample accession number SAMN18219027 and BioProject accession number PRJNA707652. The raw reads are available in the Sequence Read Archive (SRA) under accession number SRR13900999.
